# HIV epidemic drivers in South Africa: A model-based evaluation of factors accounting for inter-provincial differences in HIV prevalence and incidence trends

**DOI:** 10.4102/sajhivmed.v18i1.695

**Published:** 2017-07-28

**Authors:** Leigh F. Johnson, Rob E. Dorrington, Haroon Moolla

**Affiliations:** 1 Centre for Infectious Disease Epidemiology and Research, University of Cape Town, South Africa; 2 Centre for Actuarial Research, University of Cape Town, South Africa

## Abstract

**Background:**

HIV prevalence differs substantially between South Africa’s provinces, but the factors accounting for this difference are poorly understood.

**Objectives:**

To estimate HIV prevalence and incidence trends by province, and to identify the epidemiological factors that account for most of the variation between provinces.

**Methods:**

A mathematical model of the South African HIV epidemic was applied to each of the nine provinces, allowing for provincial differences in demography, sexual behaviour, male circumcision, interventions and epidemic timing. The model was calibrated to HIV prevalence data from antenatal and household surveys using a Bayesian approach. Parameters estimated for each province were substituted into the national model to assess sensitivity to provincial variations.

**Results:**

HIV incidence in 15–49-year-olds peaked between 1997 and 2003 and has since declined steadily. By mid-2013, HIV prevalence in 15–49-year-olds varied between 9.4% (95% CI: 8.5%–10.2%) in Western Cape and 26.8% (95% CI: 25.8%–27.6%) in KwaZulu-Natal. When standardising parameters across provinces, this prevalence was sensitive to provincial differences in the prevalence of male circumcision (range 12.3%–21.4%) and the level of non-marital sexual activity (range 9.5%–24.1%), but not to provincial differences in condom use (range 17.7%–21.2%), sexual mixing (range 15.9%–19.2%), marriage (range 18.2%–19.4%) or assumed HIV prevalence in 1985 (range 17.0%–19.1%).

**Conclusion:**

The provinces of South Africa differ in the timing and magnitude of their HIV epidemics. Most of the heterogeneity in HIV prevalence between South Africa’s provinces is attributable to differences in the prevalence of male circumcision and the frequency of non-marital sexual activity.

## Introduction

South Africa’s HIV epidemic is highly heterogeneous, with population HIV prevalence levels ranging between 5.0% in the Western Cape and 16.9% in KwaZulu-Natal in 2012.^1^ In such settings, it has been suggested that policymakers should focus HIV prevention efforts on the regions in which HIV incidence is greatest, in order to make efficient use of limited HIV resources.^2,3,4^ It is therefore important to produce robust estimates of provincial HIV prevalence and incidence.

Models may also be required to estimate HIV prevalence at district levels for the purpose of district-level treatment coverage estimation and resource allocation. Although previous studies have estimated district HIV prevalence from antenatal HIV survey data,^5^ antenatal survey data are known to be biased.^6,7,8^ It is likely that the extent of the bias differs between districts as a result of differences in the factors that account for the bias (e.g. patterns of health-seeking behaviour, contraception, epidemic stage and age distributions). It is therefore important to assess the extent of differences in antenatal bias between regions so that resource allocation is not unfairly skewed towards those districts in which the antenatal bias is greatest.

Lastly, an understanding of the factors that explain geographical differences in HIV prevalence is important in identifying epidemic drivers, which in turn is important in developing effective HIV prevention strategies. Previous studies have speculated that geographical variation in HIV prevalence within sub-Saharan Africa may be explained by differences in rates of marriage,^9^ male circumcision,^10,11,12,13^ migration,^11^ concurrency^13,14^ and other sexually transmitted infections.^15^ However, few attempts have been made to identify the factors that account for inter-provincial differences in HIV prevalence in South Africa.

This study aims to estimate HIV incidence and prevalence trends in each of South Africa’s provinces, and to identify the key epidemiological factors that account for these differences.

## Methods

The Thembisa model of the South African HIV epidemic was applied to each of the nine provinces in South Africa. Detailed descriptions of the national model^16^ and the province-specific adjustments to the national model^17^ have been published previously. Briefly, Thembisa is a combined demographic and epidemiological model, which simulates changes in the population profile and HIV disease burden over time, starting in 1985. Demographic estimates of the 1985 population profile, fertility rates, non-HIV mortality rates and migration were adapted from province-specific demographic estimates from the earlier Actuarial Society of South Africa (ASSA) 2008 model,^18^ updated to ensure consistency with the 2011 census,^19^ vital registration statistics^20,21^ and the 2010 National Burden of Disease study.^22,23^

Sexual behaviour is modelled by dividing the sexually experienced population into two broad classes: ‘high risk’ (individuals who have a propensity for concurrent partnerships and commercial sex) and ‘low risk’. Within these two classes, individuals are divided into sub-classes on the basis of their marital status and (if they are high-risk women) current engagement in commercial sex. The national assumption about the fraction of individuals who are high risk (35% for males and 25% for females) is adjusted by a multiplicative factor, which differs by province. Because of the uncertainty regarding this adjustment, a Bayesian approach is adopted, with prior distributions being specified to represent the range of uncertainty around the appropriate adjustment factor, for each province (Table 1). The mean and standard deviation of each prior distribution were chosen based on provincial differences in male reporting of concurrent partnerships^24^ and multiple partnerships in the last year.^25^ Similar multiplicative adjustments are made to national assumptions about rates of marriage, based on provincial differences in estimates of the age at first marriage.^26^

**TABLE 1:** Prior distributions

Parameter	High-risk adjustment factor†		Sexual mixing parameter‡		Condom use adjustment factor†		Initial HIV prevalence in high-risk women aged 15–49§		Antenatal bias (logit scale)†	
				
	Mean	Standard deviation	Mean	Standard deviation	Mean	Standard deviation	Mean	Standard deviation	Mean	Standard deviation
**Prior mean (SD)**										
EC	0.84	0.21	0.35	0.15	0.99	0.099	0.10	0.048	0.38	0.019
FS	1.12	0.28	0.35	0.15	1.14	0.114	0.10	0.048	0.39	0.020
GT	1.20	0.30	0.35	0.15	1.06	0.106	0.15	0.071	0.51	0.026
KZ	1.29	0.32	0.35	0.15	1.08	0.108	0.20	0.095	0.38	0.019
LP	0.83	0.21	0.35	0.15	1.04	0.104	0.10	0.048	0.36	0.018
MP	0.97	0.24	0.35	0.15	1.02	0.102	0.10	0.048	0.41	0.020
NC	0.51	0.13	0.35	0.15	0.64	0.064	0.10	0.048	0.44	0.022
NW	0.86	0.22	0.35	0.15	1.05	0.105	0.10	0.048	0.37	0.019
WC	0.61	0.15	0.35	0.15	0.74	0.074	0.10	0.048	0.49	0.025

SD, standard deviation; EC, Eastern Cape; FS, Free State; GT, Gauteng; KZ, KwaZulu-Natal; LP, Limpopo; MP, Mpumalanga; NC, Northern Cape; NW, North West; WC, Western Cape.

† , Gamma;

‡ , Beta;

§ , Uniform.

A sexual mixing parameter determines the extent of sexual contact between the high-risk and low-risk groups, with this parameter varying between 0 (no contact) and 1 (random sexual mixing).^27^ In the national model, the parameter was set to 0.47, but in initial attempts to fit the model to province-specific data, best-fitting parameter values ranged between 0.1 and 0.6 (average value of 0.35). To represent the uncertainty around the sexual mixing parameter in each province, a prior distribution was therefore assigned, with a mean of 0.35 and a standard deviation of 0.15 (Table 1).

Rates of partnership formation, coital frequency and condom use are assumed to differ by age, sex, risk group and relationship type. Rates of condom use are also assumed to change over time, in response to HIV communication programmes, and are assumed to increase following HIV diagnosis. National assumptions about probabilities of condom use are adjusted by province-specific multiplicative factors. Prior distributions are specified to represent the uncertainty around these adjustments (Table 1), with means and standard deviations being chosen based on survey estimates of differences in condom use between provinces.^1,28,29^

The model distinguishes between the ‘background’ rate of male circumcision (the rate that would be expected in the absence of campaigns to promote male circumcision as an HIV prevention strategy) and the rate of medical male circumcision (MMC) associated with campaigns promoting MMC. The annual background rate of male circumcision differs by age and province, with the age pattern being determined by assumed fractions of males circumcised during infancy, fractions of males who ever get circumcised and median ages at circumcision post-infancy. These assumptions are set to match the patterns of male circumcision in each of South Africa’s language groups in 2002^30^ and are weighted by the province-specific proportions of the population in each language group^19^ in order to obtain average provincial background rates of circumcision. Rates of MMC uptake through campaigns are assumed to be proportional to men’s probability of engaging in non-marital relationships, and are estimated from annual numbers of MMC operations reported by the Department of Health,^31,32^ distributed between provinces in proportion to numbers of uncircumcised men and in proportion to stated levels of MMC acceptability, which again differ by language.^33,34,35,36,37^

In each province, the epidemic is seeded by specifying an initial level of HIV prevalence in high-risk women aged 15–49 in 1985 and a relative level of prevalence in men aged 15–49. The uncertainty regarding the former is represented by prior distributions (Table 1). These prior distributions are determined by calculating the rate of growth in antenatal HIV prevalence over the first five survey years (1990–1994) and back-projecting the likely antenatal prevalence in 1985. Sexual transmission of HIV after 1985 is modelled based on assumed probabilities of transmission per act of unprotected sex, which differ according to age, sex, relationship type and the disease stage of the HIV-positive partner. Female-to-male transmission rates are reduced by 60% if the male partner is circumcised,^38^ and transmission rates are reduced by 90% if a condom is used. HIV transmission probabilities per act of sex are assumed to be the same across all provinces.

Untreated HIV-positive adults are stratified according to their CD4 count and HIV testing history, while treated adults are stratified according to their baseline CD4 count at antiretroviral treatment (ART) initiation and time since first ART initiation. Assumptions about rates of CD4 decline and HIV mortality are the same for all provinces. However, assumptions about rates of HIV testing and ART initiation differ by province, based on reported numbers of HIV tests performed^39^ and numbers of patients who are on ART by province.^40^ The model also allows for differences between provinces in rates of antenatal HIV testing,^41^ uptake of prophylaxis against mother-to-child transmission, breastfeeding by HIV-positive mothers and uptake of polymerase chain reaction (PCR) screening after birth.^42,43^

Each provincial model is fitted to province-specific HIV prevalence data from the 1990–2013 antenatal surveys^44^ and household surveys conducted in 2005, 2008 and 2012.^1^ A detailed description of the method used to calculate the likelihood function is provided in the Online Appendix. Because the antenatal surveys represent only women using public antenatal facilities, and because of other biases, an antenatal bias parameter is specified for each province to represent the average difference between the true HIV prevalence in pregnant women and that measured in the survey. Prior distributions are specified to represent the uncertainty around these biases (Table 1), with the prior means and standard deviations being set based on the biases estimated when fitting the national model, adjusted to take into account provincial differences in the fraction of adults who are members of private medical schemes.^29^ Posterior estimates of the best-fitting parameter values were calculated using Incremental Mixture Importance Sampling.^45^

## Results

**FIGURE 1:**
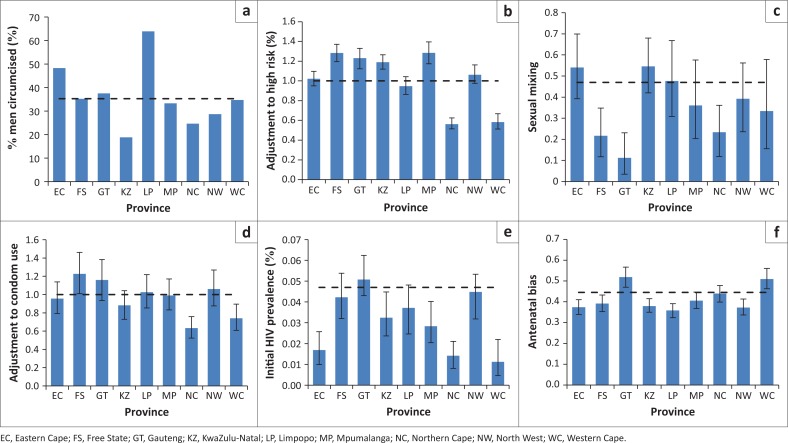
Male circumcision rates and posterior estimates of sexual behaviour parameters, initial HIV prevalence and antenatal bias. Panel (a) shows the modelled prevalence of male circumcision in men aged 15–49 years in 2000 (prior to MMC promotion campaigns); (b) Multiplicative adjustment to high risk proportion; (c) Sexual mixing parameter; (d) Multiplicative adjustment to condom usage; (e) Initial HIV prevalence in women aged 15–49 years (initial HIV prevalence in high-risk women multiplied by the fraction of women in the high-risk group); (f) Antenatal bias (on logit scale). Panels (b)–(f) show posterior means of the input parameters for which prior distributions have been specified (Table 1), and error bars represent the 95% confidence intervals from the posterior distributions. In all panels, the dashed line represents the national average.

Differences in the provincial assumptions about male circumcision led to the modelled fraction of 15–49 year old men who were circumcised in 2000 varying between 19% in KwaZulu-Natal and 64% in Limpopo (Figure 1a). After fitting the model to HIV prevalence data, a number of the other parameters were also found to differ substantially between provinces. The high-risk adjustment factor was significantly below the national average in Northern Cape (0.56, 95% CI: 0.51–0.63) and Western Cape (0.58, 95% CI: 0.51–0.67), but significantly above the national average in Free State, Gauteng, KwaZulu-Natal and Mpumalanga (Figure 1b). The sexual mixing parameter ranged between 0.11 (95% CI: 0.04–0.23) in Gauteng and 0.55 (95% CI: 0.42–0.68) in KwaZulu-Natal (Figure 1c). Condom usage was significantly below the national average in Northern Cape and Western Cape, but not significantly different from the national average in other provinces (Figure 1d). Initial HIV prevalence in women aged 15–49 in 1985 was highest in Gauteng (0.051%, 95% CI: 0.043%–0.062%) and lowest in the provinces in the southern and western parts of the country (Figure 1e). Finally, the extent of the antenatal bias was greatest in Gauteng and Western Cape (Figure 1f). Prior and posterior estimates of each parameter are compared as shown in Online Appendix Figure 1.

**FIGURE 2:**
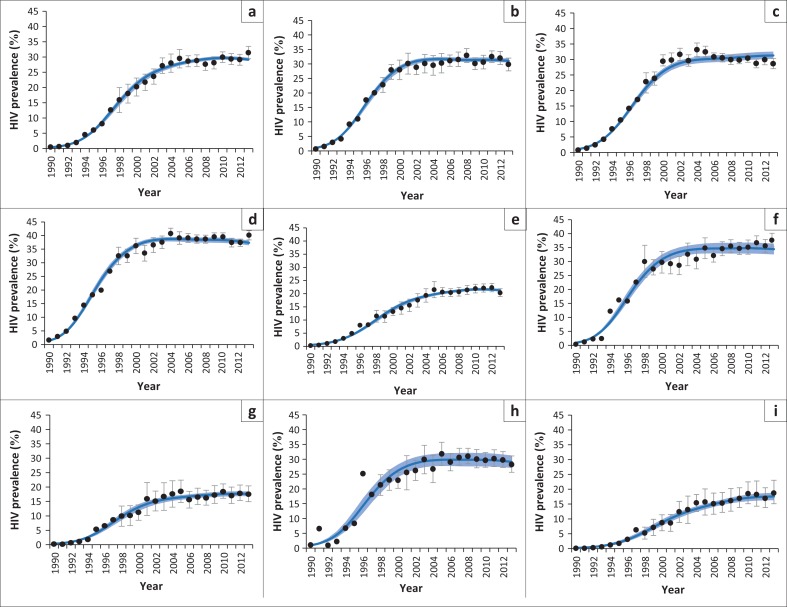
HIV prevalence levels in pregnant women attending public antenatal clinics: (a) Eastern Cape; (b) Free State; (c) Gauteng; (d) KwaZulu-Natal; (e) Limpopo; (f) Mpumalanga; (g) Northern Cape; (h) North West; (i) Western Cape. Dark blue lines represent posterior means and shaded light blue areas represent posterior 95% confidence intervals (model estimates have been adjusted to reflect the modelled antenatal bias). Dots represent antenatal survey estimates (95% confidence intervals for survey estimates prior to 1998 are not shown, as the reported confidence intervals did not account for survey design effects).

Figure 2 shows that the model estimates of antenatal HIV prevalence over the 1990–2013 period are generally in close agreement with the survey data. However, some of the early antenatal surveys in North West and Mpumalanga produced erratic trends, with the result that there are wide confidence intervals around the model estimates. In addition, the model tends to under-estimate HIV prevalence in Gauteng in the early 2000s. In sensitivity analyses that excluded antenatal data collected prior to 1997 (before the introduction of standard sampling protocols), average model estimates of HIV prevalence over the 1997–2013 period were virtually unchanged, but confidence intervals were substantially narrower in North West and Mpumalanga (Online Appendix). The model fit to the Gauteng data was not improved in sensitivity analyses that considered different priors on the sexual behaviour parameters (Online Appendix).

**FIGURE 3:**
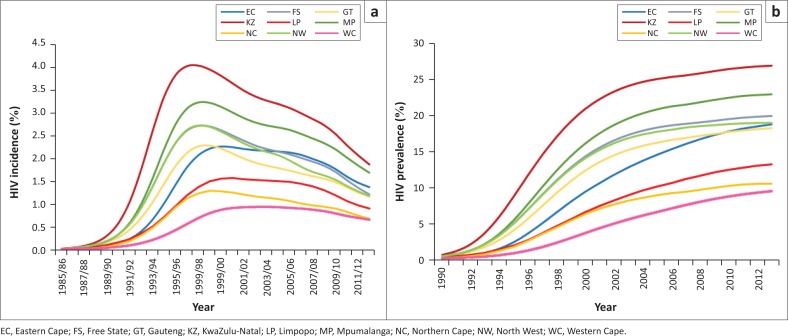
HIV incidence: (a) and prevalence (b) trends in 15–49-year-olds. Lines represent posterior means (95% confidence intervals not shown).

HIV incidence trends differed substantially by province (Figure 3a). Incidence in 15–49-year-olds peaked in KwaZulu-Natal in 1997–1998 at 3.97% per annum (95% CI: 3.81%–4.13%) and subsequently declined to 1.83% (95% CI: 1.71%–1.93%) in 2012–2013. In contrast, incidence in the Western Cape peaked in 2003–2004 at 0.92% (95% CI: 0.84%–0.99%) and declined to 0.64% (95% CI: 0.53%–0.75%) by 2012–2013. HIV prevalence trends reflect similar inter-provincial differences (Figure 3b); by mid-2013, HIV prevalence in 15–49-year-olds varied between 9.4% (95% CI: 8.5%–10.2%) in Western Cape and 26.8% (95% CI: 25.8%–27.6%) in KwaZulu-Natal. Although HIV prevalence has been steadily increasing in all provinces over the last five years, the pace of increase has been greater in Western Cape, Limpopo and Eastern Cape, reflecting the slower pace of HIV incidence decline in these provinces.

**FIGURE 4:**
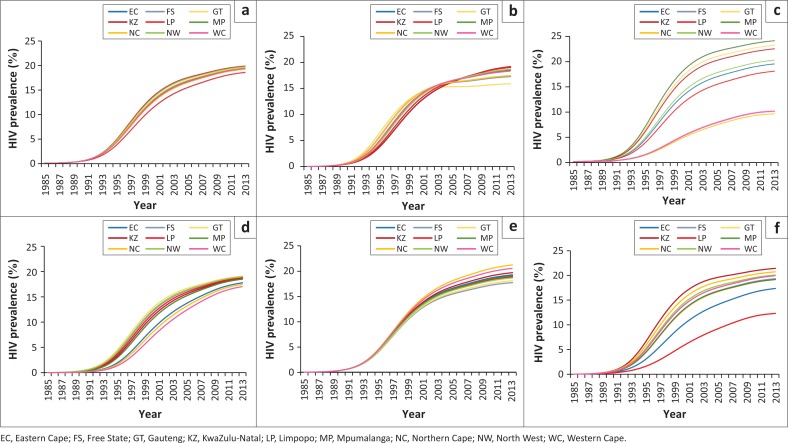
Effect on adult HIV prevalence (15–49 years) in the national HIV model of substituting province-specific parameter values: (a) Substituting provincial marriage rates; (b) Substituting provincial sexual mixing parameters; (c) Substituting provincial high risk proportions; (d) Substituting provincial initial HIV prevalence levels; (e) Substituting provincial rates of condom use; (f) Substituting provincial male circumcision rates. For panels (b)–(e), province-specific parameters substituted into the national model are the posterior means shown in Figure 1.

To assess the relative importance of different parameters in explaining inter-provincial prevalence differences, the parameters estimated in each provincial model were substituted into the national model in a series of one-way sensitivity analyses (Figure 4). HIV prevalence in 2013 in 15–49-year-olds varied between 18.2% when substituting the Free State marriage rates into the model and 19.4% when substituting the KwaZulu-Natal marriage rates into the model (Figure 4a). Prevalence varied between 15.9% when substituting the Gauteng sexual mixing parameter into the model and 19.2% when substituting the KwaZulu-Natal sexual mixing parameter into the model (Figure 4b). In contrast, prevalence was highly variable when substituting province-specific high-risk adjustments, ranging from 9.5% when using the Western Cape parameter to 24.1% when using the Mpumalanga parameter (Figure 4c). Although provincial differences in initial HIV prevalence (in 1985) accounted for much variation in prevalence during the 1990s and early 2000s (Figure 4d), prevalence in 2013 was relatively insensitive to the initial prevalence, ranging between 17.0% when substituting the Western Cape initial prevalence and 19.1% when substituting the Gauteng initial prevalence (Figure 4d). In contrast, provincial differences in condom use accounted for little variation in prevalence during the 1990s, but accounted for more variation in prevalence in 2013: prevalence in this year varied between 17.7% when substituting the Free State condom use and 21.2% when substituting Northern Cape condom use. Finally, prevalence was very sensitive to the assumed levels of male circumcision prior to MMC promotion, varying between 12.3% when substituting the Limpopo parameters and 21.4% when substituting the KwaZulu-Natal parameters into the national model.

## Discussion

This analysis confirms the heterogeneous distribution of HIV within South Africa and advances our understanding of the factors that account for this heterogeneity. Most of the current inter-provincial variation in HIV prevalence in South Africa is attributable to two factors: differences in the prevalence of male circumcision and differences in the fraction of the population in the high-risk group. Because the model assumes that rates of marriage are the same in the high-risk and low-risk groups, but rates of non-marital sex (short-term relationships and sex worker–client contacts) differ between high-risk and low-risk, the provincial differences in the high-risk fraction are equivalent to differences in the frequency of non-marital sex. Our findings of relatively low rates of non-marital sex in Western Cape and Northern Cape are consistent with the findings of sexual behaviour surveys, which show that the fraction of men reporting multiple or concurrent partnerships is lowest in these two provinces.^24,25^

Rates of male circumcision are highly variable between South African ethnic groups. The Pedi and Venda ethnic groups, which account for the majority of the population in Limpopo, have high rates of circumcision, and circumcision typically occurs in early adolescence.^14,30^ The Xhosa, which comprise 79% of the Eastern Cape population,^19^ also have high rates of male circumcision, but circumcision occurs at later ages than in other ethnic groups,^30,33^ with the result that there is less HIV prevention benefit. The Zulu, which account for 78% of the population in KwaZulu-Natal, had the lowest fraction of men circumcised in 2002 (14.5%),^30^ which partially accounts for the severity of the HIV epidemic in this province.

Ethnicity may also be an important factor explaining provincial differences in sexual mixing patterns. It is interesting to note that the sexual mixing parameter is lowest in Gauteng, the most ethnically heterogeneous province, and is highest in Eastern Cape and KwaZulu-Natal, which are ethnically relatively homogeneous (Figure 1c). Although the sexual mixing parameter is defined to represent the extent of mixing between high-risk and low-risk groups, the model-fitting procedure may effectively be identifying mixing in relation to other dimensions of HIV risk (such as ethnicity and socioeconomic status). To the extent that individuals tend to be ethnically and socioeconomically homogamous,^46,47,48^ one might expect to observe less sexual mixing in the provinces in which there is greater ethnic/socioeconomic heterogeneity. This is important because mathematical models suggest that HIV epidemics develop differently depending on sexual mixing patterns: when there is little sexual mixing between high-risk and low-risk groups, HIV spreads more rapidly at first but levels off at a lower rate (Figure 4b),^49,50,51^ and the greater degree of heterogeneity in HIV risk means that interventions have less of an impact on HIV incidence.^52^ Thus the relatively rapid levelling off in HIV prevalence seen in Gauteng is likely to be because of the low level of sexual mixing in this province rather than any unusually successful HIV intervention.

Differences in the timing of peak HIV incidence are largely explained by differences in the prevalence of HIV in 1985, which is a proxy for the age of the epidemic. Consistent with earlier analyses,^53^ we find that the HIV epidemic started later in Eastern Cape, Northern Cape and Western Cape than the other provinces (Figure 1e), and HIV incidence therefore peaked later in these provinces (Figure 3a). However, in recent years differences in epidemic timing account for relatively little inter-provincial variation.

This analysis suggests that antenatal bias differs substantially between provinces, being most extreme in the provinces in which use of private healthcare is greatest. Caution is therefore required when using antenatal HIV prevalence data to draw conclusions about differences in HIV burden between provinces or districts. Ideally, such comparisons should rely on HIV prevalence data from household surveys, but the cost of such surveys and the difficulty in obtaining adequately precise estimates for small geographical units often make this impractical.

There have been few previous attempts to model the role of different factors in explaining inter-provincial HIV variation within South Africa. The ASSA models explained differences in HIV prevalence across provinces primarily in terms of provincial differences in initial HIV prevalence, proportions of the population in different risk groups and population group profile (for example, the low prevalence of HIV in the Western Cape was partly explained by the relatively small proportion of black South Africans living in this province).^54^ However, the models did not consider directly the role of factors such as male circumcision or marriage. Another modelling study noted that provincial differences in measures of HIV incidence and epidemic growth did not correlate strongly with the prevalence of male circumcision, and hence concluded that male circumcision was not a significant driver to HIV spread in South Africa.^55^ However, this study did not consider the role of provincial differences in sexual behaviour patterns. Understanding reasons for inter-provincial differences in HIV prevalence requires a model that is sophisticated enough to simulate each of the major epidemic drivers simultaneously.

A strength of this analysis is that it relies on a parsimonious model-fitting procedure, with only a few key parameters being used to explain the differences in HIV prevalence between provinces. Unlike the approach in other HIV model-fitting studies,^56,57^ these parameters relate to observable measures of behaviour and HIV risk, allowing an understanding of the factors that are most influential in driving HIV. However, the parsimony of the model-fitting procedure is also a limitation, as there may be important epidemic drivers that we have not been able to identify because of the simple model structure. For example, drug and alcohol use may be important determinants of HIV risk behaviour,^58,59^ but are not currently considered in the model. There are also variables that might not be important in explaining inter-provincial HIV differences, but which are nevertheless important as epidemic drivers. For example, differences in marriage rates account for relatively little variation in HIV prevalence between provinces (Figure 4a), but this may be because marriage rates in South Africa are uniformly low in comparison to the rates in most other African countries.^9^

Another limitation associated with the relatively parsimonious model-fitting procedure is that the model fit to the antenatal survey data is not always as good as might be hoped for, especially in the case of Gauteng. This implies that there is some uncertainty around the HIV incidence estimates, particularly in the most recent years. Incorporating other data sources, such as recorded death data, in the calibration procedure could potentially lead to more precise estimates. However, recorded death data have not been used in the calibration of the provincial models because of current uncertainty regarding provincial differences in the fractions of deaths that are recorded,^60^ and the possibly substantial fraction of individuals who die outside of the province in which they are normally resident.^60,61^

Another limitation of this study is that uncertainty regarding the prevalence of male circumcision is not considered in the model-fitting procedure. Assumptions about the prevalence of male circumcision were considered more robust than the assumptions about sexual behaviour, the former having been validated using data from various nationally representative surveys.^62,63^ There is also uncertainty regarding relative levels of risk behaviour in circumcised and uncircumcised men. Consistent with most published studies,^64,65,66,67^ the model assumes that male circumcision is not associated with any change in HIV risk behaviour. However, one South African study found a univariate association between traditional male circumcision and reduced HIV risk perception, as well as a univariate association between MMC and reporting of multiple partnerships.^62^ Another Cape Town study found that HIV risk behaviours in traditionally circumcised men were related to knowledge of the protective effects of male circumcision.^68^ However, even if it is true that increased awareness of the protective effects of male circumcision is leading to increased risk behaviour in circumcised men, this does not diminish the importance of male circumcision in explaining inter-provincial HIV prevalence differences in the period up to 2005, before the protective effect of male circumcision was widely known.

Although this analysis does not consider provincial differences in HIV testing and ART coverage as factors explaining differences in HIV prevalence, we have shown that levels of HIV diagnosis are similar across provinces,^17^ and thus they would be unlikely to account for HIV prevalence differences. ART reduces HIV incidence, but the impact that this has on HIV prevalence is completely offset by the impact on mortality in the short term.^69^ The net effect of provincial differences in ART access on provincial HIV prevalence is therefore also likely to be relatively small.

## Conclusion

The conclusion that the low prevalence of male circumcision and high prevalence of multiple/concurrent partnerships are important in driving the high HIV prevalence in southern Africa, is not new.^13^ However, advances in new HIV prevention and treatment strategies have meant that these factors have received relatively less attention in recent HIV prevention debates. It is important that these epidemic drivers are not neglected in the push towards the ‘90-90-90’ targets,^70^ and that HIV communication programmes continue to promote male circumcision and risk awareness in the context of non-marital relationships.
